# 50 Years of Stereoblindness: Reconciliation of a Continuum of Disparity Detectors With Blindness for Disparity in Near or Far Depth

**DOI:** 10.1177/2041669517738542

**Published:** 2017-11-16

**Authors:** Reinder Dorman, Raymond van Ee

**Affiliations:** Cognitive and Systems Neuroscience Group, Swammerdam Institute for Life Science, University of Amsterdam, The Netherlands; Donders Institute for Brain, Cognition and Behavior, Radboud University, Nijmegen, The Netherlands; 198328 Donders Institute for Brain, Cognition and Behavior, Radboud University, Nijmegen, The Netherlands; Department of Brain and Cognition, University of Leuven, Belgium; Department of Brain, Behavior and Cognition, Philips Research, Eindhoven, The Netherlands

**Keywords:** stereopsis, stereoblindness, three-dimensional perception, neural mechanisms, disparity sensitivity

## Abstract

Whitman Richards (1932–2016) discovered some 50 years ago that about 30% of observers from the normal population exhibit stereoblindness: the disability to process binocular disparities in either far or near depth. We review the literature on stereoblindness entailing two insights. First, contemporary scholars in stereopsis undervalue the comprehension that disparity processing studies require precise assessments of observers’ stereoblindness. We argue that this frequently leads to suboptimal interpretations. Second, there is still an open conundrum: How can the established finding that disparity is processed by a continuum of detectors be reconciled with the disability of many observers to process a whole class of far or near disparities? We propose, based upon integration of literature, that an asymmetry between far and near disparity detection at birth—being present for a variety of reasons—can suppress the typical formation of binocular correlation during the critical period for the development of stereopsis early in life, thereby disabling a whole class of far or near disparities.

## Introduction

It is some 50 years ago that Whitman Richards (1932–2016) and colleagues in the field of stereopsis started to systematically investigate individual subject differences. One of the achievements in this field that is inextricably linked to Richards concerns stereoblindness. Richards experimentally discovered that about 30% of subjects, with apparently normal vision, lack the ability to process binocular disparities specifically in either far or near depth relative to the fixation depth (Richards, 1971).

Stereoblindness has caused quite some discussion even a long time after its discovery. Although nowadays stereoblindness is replicated for a wide range of stereoscopic stimuli, the experimental importance of stereoblindness is still frequently undervalued by scholars in stereopsis. Here it is key to appreciate that for proper studies on disparity processing, and the role of stereoblindness in data interpretation, one is bound to use brief presentation durations—in the order of 100 ms—to prevent fixational eye movements in depth to compensate for a lack of far or near disparity sensitivity. We pronounce this brief presentation issue at the outset of this review because later we discuss that this is where researchers can be incautious.

Next to the issue of the role of fixational eye movements, there has been quite some discussion on the neurophysiological underpinning of stereoblindness. Richards (1970) suggested that depth perception was processed by three distinct disparity detection pools: a pool for large disparities in front of the fixation plane (i.e., *near* depth), for behind the fixation plane (*far* depth), and for small disparities at the fixation plane (*zero* depth). The three-pool hypothesis was inspired by findings in colourblindness. In analogy with colourblindness, Richards (1970) proposed that a genetic deficit could cause the lack of one of the three disparity detection pools. While early neurophysiological work seemed to be consistent with the three-pool hypothesis, later work, both in psychophysics and in neurophysiology, demonstrated that disparity processing involves a continuum of many overlapping disparity detectors (reviewed in Landers & Cormack, 1997). This leaves us with the conundrum that, on the one hand, there is a continuum of many overlapping disparity detectors, and on the other hand, many observers are blind for a complete class of near or far disparities.

In the next sections, we will review stereoblindness studies in psychophysics and electrophysiology, respectively. Finally, we discuss how studies in binocular correlation during a critical period for the development of stereopsis early in life can be used to explain how a continuum of disparity detectors in normal stereopsis could be reconciled with the finding that many observers are blind for a whole class of binocular disparity.

## Psychophysics

**Figure 1.**
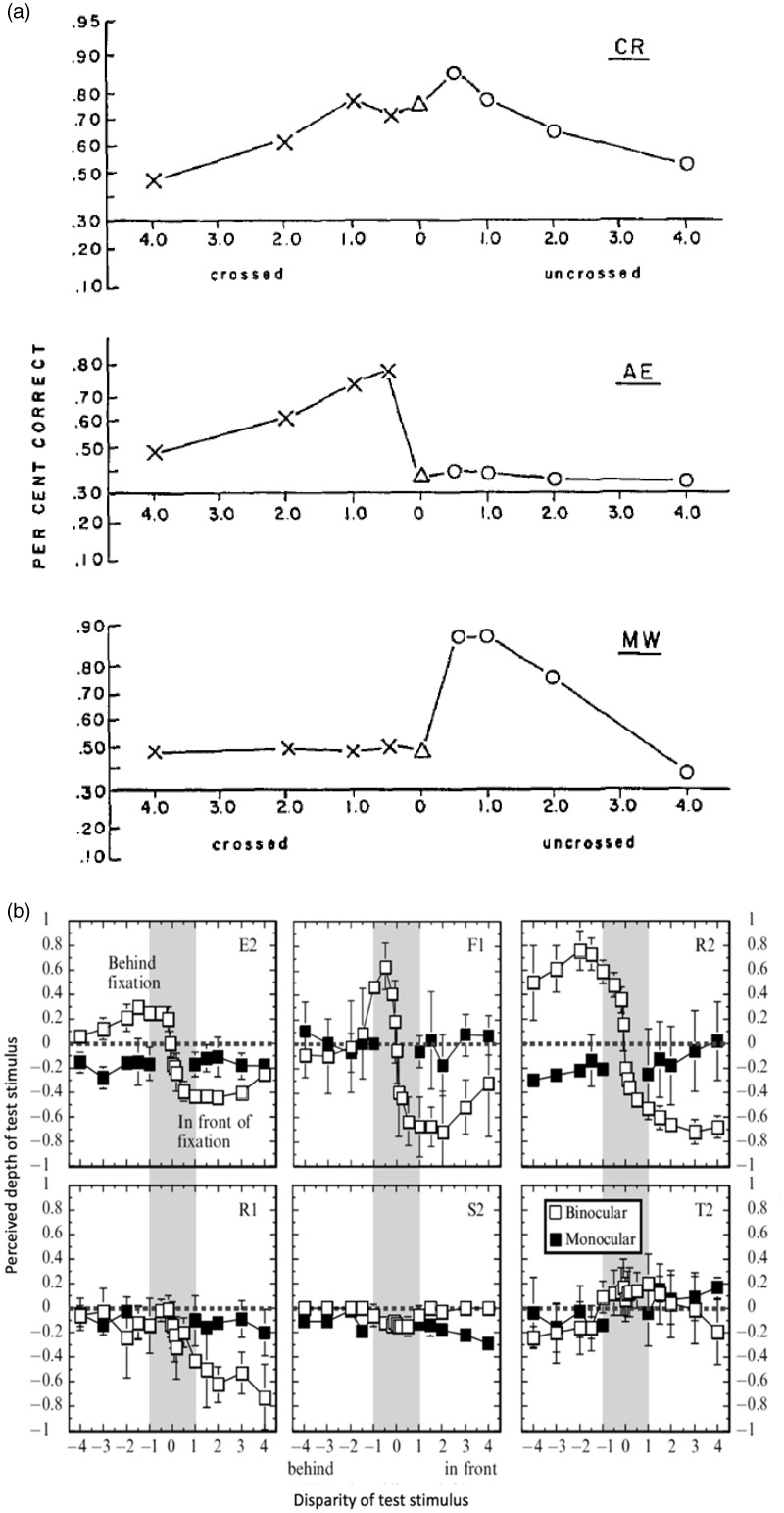
Psychophysical data showing that a subset of subjects is not able to correctly perceive certain disparities. (a) A three-alternative depth discrimination task shows normal subjects (CR) performing above chance level, and two stereoblind subjects unable to detect far (AE) and near (MW) disparity. From Richards (1971), with permission. (b) In a contemporary setup, van Ee and Richards (2002) showed similar results in a depth estimation task. Normal subjects (E2, F1, and R2), show differing perceived magnitudes of depth and correctly interpret its sign. Stereoblind subjects fail to correctly perceive depth: not perceiving far disparity (R1), perceiving small disparities (−1 to 1°) only as in front (S2), or frequently reversing the sign of depth (T2). From van Ee and Richards (2002), with permission.

Richards studied stereoblindness using a briefly flashed (80 ms) bar stimulus, ranging from −4 to +4° disparity relative to fixation. A distribution of the subjects’ (*n* = 75) three-alternative forced-choice discriminations (*near*, *far*, or *on fixation*) indicated that a considerable portion of the subjects perceived bars that were veridically either closer or behind the fixation point, as being *on fixation* (Richards, 1970). Individual data are represented in Figure 1(a). Normal subjects (e.g., CR) responded correctly in almost all trials for small disparities and performed above chance for larger disparities (Richards, 1971). In contrast, about 30% of subjects were not able to perceive depth for bars either closer or behind fixation (e.g., AE and MW).

To replicate the traditional stereoblindness test using modern stimulus generation techniques, van Ee and Richards (2002) developed a planar and a volumetric stereoblindness test. Figure 1(b) shows depth matching results from the planar test. The top row displays three normal subjects, and the bottom row shows stereoblind subjects. Subject F1 shows good depth perception, correctly perceiving differences between small magnitudes of disparity (F1, gray area). Subject R1 perceives near depth but not far depth. Subject S2 does not perceive far depth, and the magnitude of perceived depth is small for near depth. Subject T2 sporadically perceives reversed depth perception.

**Figure 2.**
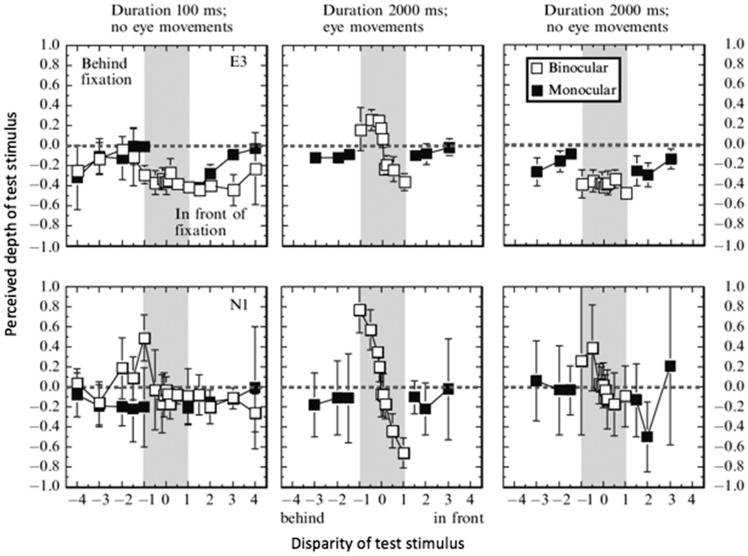
Correct fixation is crucial to study stereoblindness. In a depth estimation task, two stereoblind subjects (different rows, E3 and N1) show incorrect depth (left panels), improving drastically with a 2000 ms stimulus and free eye movements (middle panels). When eye movements were restricted during the longer stimulus duration (right panels), performance was again poor. From van Ee and Richards (2002), with permission.

We have mentioned earlier that stimulus duration is of cardinal importance. When the stimulus is not briefly flashed, subjects can employ fixation-in-depth strategies to mask stereoblindness for targets that are either in front or behind the fixation depth. A subject lacking near depth perception, but not far depth perception, could put their fixation closer than the target, in which case a near disparity would become a far disparity and the depth of the target relative to the fixation point would become visible. Data of van Ee and Richards (2002) show how stereoblind subjects greatly improve in performance once the stimulus is presented longer and eye movements were encouraged (Figure 2). However, when fixation was to be kept strictly on the fixation point for the longer duration, stereoblind subjects again performed poorly, as they did on the briefly presented stimulus. It can be concluded that eye movements, not stimulus duration, is the critical factor in revealing stereoblindness.

To generalize the conventional planar bar stereoblindness test to volumetric stimuli, van Ee and Richard (2002) exposed subjects to a flashed random array of multiple bars subtending a static volume in depth. They found that stereoblind subjects were unable to perceive depth in this volumetric stimulus. Moreover, the planar and volumetric tests predicted each other’s outcome. The conventional planar stereoblindness test has also been conducted in a more sophisticated three-dimensional (3D) stimulation set up (Kooi, Dekker, van Ee, & Brouwer, 2010). In this study, a second moving monitor, reflected by a half-silvered mirror, was used to display the depth stimulus, while the fixation point was on a static monitor. This minimized conflicting monocular depth cues, like lenticular accommodation or pixel size. Although the 3D display enhanced perceived depth and was reported to be more comfortable, the stereoblind subjects still lacked normal depth perception, as in the previous studies.

Multiple psychophysical studies (Birch, Gwiazda, & Held, 1982; Breitmeyer, Julesz, & Kropfl, 1975; Finlay, Manning, Dunlop, & Dewis, 1989; Herring & Bechtoldt, 1981; Manning, Finlay, Neill, & Frost, 1987; Patterson et al., 1995; Regan, Erkelens & Collewijn, 1986; Schor & Wood, 1983) have reported on asymmetries in sensitivity for far and near disparities (see also reviews in Landers & Cormack, 1997; Mustillo, 1985; Regan, Frisby, Poggio, Schor, & Tyler, 1990). For example, developmental work showed that infants develop near stereopsis prior to far stereopsis (Birch et al., 1982). In the adult, it has been reported that there are different spatiotemporal response functions for far and near disparities (Finlay et al., 1989; Manning et al., 1987; Patterson et al., 1995; Schor & Wood, 1983).

In their thorough psychophysical experimental studies, Cormack, Stevenson, and Schor (1973) also found asymmetric sensitivity for far and near disparities. They convincingly demonstrated that their experimental psychophysical results could be explained by disparity processing consisting of a continuum of disparity detectors, as opposed to the three-pool disparity processing (reviewed in Landers & Cormack, 1997). This work brings us to the neurophysiological underpinnings of stereoblindness.

## Electrophysiology

One reason why stereoblindness caused discussion even a long time after its discovery is the neurophysiological basis. The population distribution results from Richards’ first experiment were consistent with three-pool disparity processing. This became influential quickly but became debated in the nineties.

**Figure 3.**
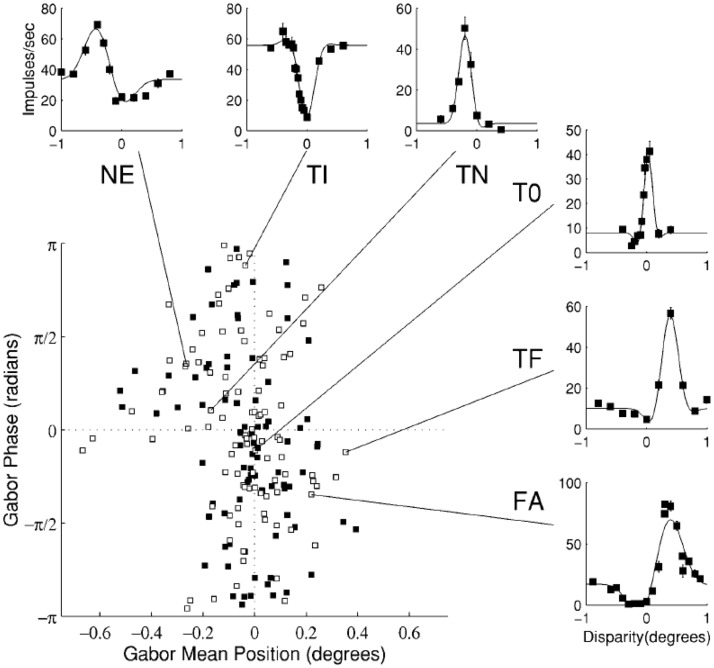
Neurons in V2 showing a continuum of responses to disparity, modelled by Gabor functions (see Anzai, Ohzawa, & Freeman, 1999, for details). The mean position (*x* axis) indicates preferred disparity, and the phase (*y* axis) determines the symmetry of the response curve. A wide range of responses can be identified, among which the several classes of Poggio et al. (insets). From Prince et al. (2002), with permission. NE = near cell, TI = tuned inhibitory, TN = tuned near, T0 = tuned zero, TF = tuned far, FA = far cell.

**Figure 4.**
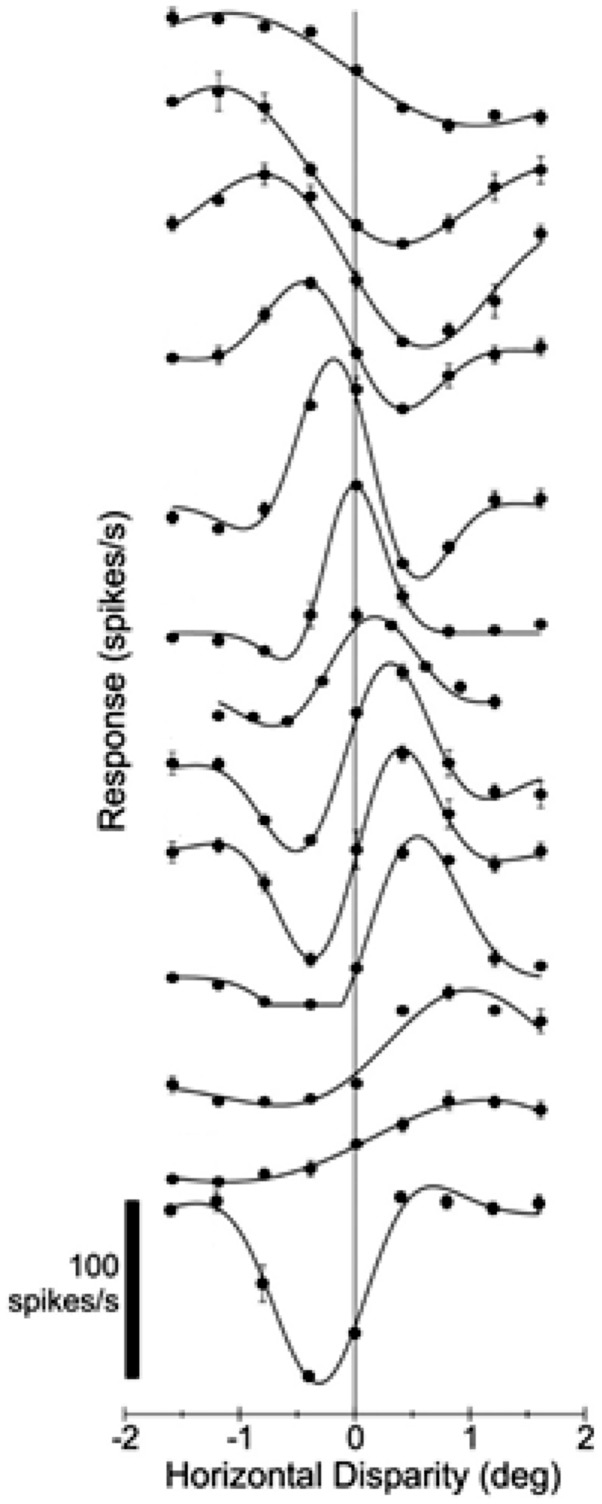
Neurons in MT show a continuum of responses to disparities, much as in V2. From DeAngelis and Uka (2003), with permission.

An early electrophysiological study by Poggio and Fischer (1977) adopted Richards’ three-pool theory to explain the observed selective near and far types of disparity neurons. Although at the time it was naturally to pronounce that these asymmetric responses in depth coincide with Richards’ theory on distinct mechanisms for far and near depth perception, Poggio and Fischer’s results were not sufficient to convincingly validate the presence of only two separate mechanisms for far and near disparity. Indeed, later more precise disparity processing categories were reported (Poggio, Gonzalez, & Krause, 1988). Since then neurophysiological results favoured a continuum of disparity sensitivity in cat (DeAngelis, Ohzawa, & Freeman, 1991; Freeman & Ohzawa, 1990; Ohzawa, DeAngelis, & Freeman, 1996) and in macaque (Prince, Cumming, & Parker, 2002). Prince et al. (2002) recorded hundreds of disparity selective neurons from the lower visual cortex of the awake macaque. They used long duration (2000 ms) trials but were careful to explicitly verify that fixation was kept within the required fixation window. Their data demonstrated that disparity sensitive neurons subtended a continuum (Figure 3). DeAngelis and Uka (2003) used comparable stimulation as Prince et al. (2002) and also found a continuum of disparity response properties in higher tier visual areas (Figure 4). These results can explain the proposed far- and near disparity tuned cells found by Poggio et al. as cells broadly tuned to large disparities. In a more recent awake macaque study, Anzai, Chowdhury, and DeAngelis (2011) found similar disparity receptive fields in motion-disparity processing areas, using constant fixation, supporting a continuum.

Results from monkey functional magnetic resonance imaging (Chen, Lu, & Roe, 2008), as well as from two-photon-calcium imaging in cat (Kara & Boyd, 2009), add to the evidence that disparity sensitive clusters are organized in a continuum from far to near. Chen et al. (2008) tested anesthetized monkey with the eyes stabilized with eye rings; in Kara and Boyd (2009), eye movements were assumed to be absent based upon stability of the cortical map.

These neurophysiology findings complement the psychophysical work reviewed in the previous section. We already mentioned that psychophysical studies, while experimentally finding asymmetric sensitivity for far and near disparities, demonstrated that disparity processing is reflected as a continuum (Landers & Cormack, 1997). Indeed, there is a considerable body of psychophysical modelling work that is consistent with a continuum in disparity tuning (Cormack et al., 1993; Landers & Cormack, 1997; Lehky & Sejnowski, 1990; Stevenson, Cormack, Schor, & Tyler, 1992; Patterson et al., 1995). To be more precise, Landers and Cormack (1997) suggested depth per se was the first component in disparity processing. The magnitude of depth would be the second, and the third would be a computation of the sign of depth. Errors in the sign computation, then, could lead to stereoblindness. In a similar vein, Poggio (1995) has suggested that the three pools can coexist with a continuum of disparity tuned neurons. Thus, the pools as defined by Richards can be responsible for a sign-error in coarse qualitative estimates of depth early in disparity processing. A later finding concerning phase- and position-shift disparity detectors suggests, however, that disparity sensitivity is immediately sign selective and continuous (Anzai, Ohzawa, & Freeman, 1999).

## Underlying Mechanism of Stereoblindness

Richards’ seminal psychophysical findings on stereoblindness were well in line with a study on binocular development in kittens with artificially induced squint (Hubel & Wiesel, 1965). Hubel and Wiesel reported that these kittens lacked binocular neurons, attributed to a lack of binocular correlation during the critical period for the development of stereopsis. In support, Movshon, Chambers, and Blakemore (1972) and Mitchell and Ware (1974) have reported that stereoblind subjects show a dramatic reduction in the extent of interocular transfer, attributed to the lack of binocular neurons. Jones (1977) studied oculomotor responses in 30 individuals and reported that the presence of vergence anomaly was always associated with the occurrence of stereoblindness. We interpret Jones’ findings as that stereoblindness could be caused by a disability in making correct fixational eye movements in depth early in life, in turn, causing a lack of exposure to binocular correlation during the critical period for the development of stereopsis.

It has been suggested that stereoblindness can also have a different origin. For example, it may be possible that the lack of binocular neurons may be genetically endowed. Congenital anomalies of visual cortical physiology are in fact known to occur in some animals of which the Siamese cat provides the best-known example (Cool & Crawford, 1972; Hubel & Wiesel, 1971).

**Figure 5.**
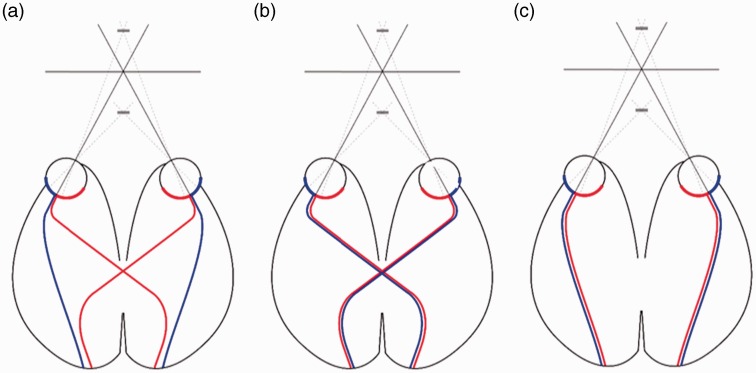
Schematic of the different hemiretinas’ axons traversing through the optic chiasm to right and left thalamus and, subsequently, cortex. Note how the different depth stimuli fall on the hemiretinae and the corresponding hemisphere. (a) Illustration of the normal situation. (b) Illustration of the total decussation, similar to decussation seen in albinism. (c) Illustration of the total lack of decussation, as in achiasmatic syndrome.

Finally, we speculate about a mechanism based on misrouting in the optic chiasm. In the optic chiasm, axons from the temporal hemiretinae traverse to the ipsilateral cortex, and axons from the nasal hemiretinae decussate, to end in the contralateral visual cortex. For a central stimulus, a near stimulus lands on both temporal hemiretinae, and a far stimulus lands on both nasal retinae (Figure 5(a)). Chiasmatic misrouting has been identified in several conditions. In human albinism and Siamese cats, the temporal hemiretinae erroneously decussates to end in the contralateral hemisphere (Hoffmann, Tolhurst, Moore, & Morland, 2003; Kalil, Jhaveri, & Richards, 1971; Figure 5(b)). Likewise, in achiasmatic syndrome, there is no decussation of the nasal hemiretinae (Apkarian, Bour, Barth, Wenniger-Prick, & Verbeeten, 1995; Figure 5(c)). These conditions would leave one side of the hemiretinae to be processed normally, while the affected hemiretinae ends in the wrong hemisphere. In such cases, one depth sign could be processed normally, while the other is misrouted, and thus impaired. Indeed, chiasmatic misrouting shows defective stereopsis (Prieur & Rebsam, 2016), but, unfortunately a differentiation between far or near sensitivity in albinism or achiasmatic syndrome has not yet been investigated. It is interesting to consider to what extent it may cause an asymmetry between near and far disparity sensitivity.

## Discussion

Scholars performing stereopsis experiments know from experience that there is a considerable number of subjects who is unable to perceive depth from a whole class of disparities either in front or behind fixation. Indeed, a significant portion (estimated by Richards [1970] at 30%) of the population exhibits anomalous behaviour in stereoblindness tests.

Just as the use of colourblind subjects in colour-vision studies has advanced our understanding of the underlying mechanisms of colour processing, it was Richards’ hope that the wider use of stereoblindness tests would give us fundamental insights into the underlying mechanisms of disparity processing. Inspired by this line of thought, van Ee (2003) investigated to what extent binocular matching is facilitated by motion in both stereoblind and normal subjects when they are required to estimate the perceived depth of a 3D stimulus that contains excessive binocular matching candidates (van Ee & Anderson, 2001). The volumetric stereoblindness test (van Ee & Richards, 2002) was developed to bridge Richards’ traditional planar stereoblindness test to 3D scenes. The main learning from the experimental study of van Ee (2003) is that interindividual subject differences in the 3D stereoscopic matching task (the extent to which binocular matching is facilitated by motion) can be explained by the subjects’ performance in the volumetric stereoblindness test. Without the results of the stereoblindness test, the results reported in van Ee (2003) would not have made sense.

Considering that a host of perceptual properties are found to be correlated with an asymmetry in near and far disparity processing (reviewed in Mustillo, 1985; Regan et al., 1990), it is surprising that tests for depth perception with stimuli of varying disparity have not routinely been used as a basis for correlating individual differences in stereoscopic information processing. A possible reason is that stereoblindness has been regarded as a *transient* phenomenon that shows up only when targets are presented for a short duration (Newhouse & Uttal, 1982). However, in Newhouse and Uttal’s experiment, the observation period was 2 minutes, and eye movements were unrestricted, which enabled the subject to put a near stimulus in the far region (and vice versa) to mask stereoblindness. This means that their experiment did not test stereoblindness. The results in Figure 2 indicate that eye movement, not stimulus duration, is the critical factor in revealing stereoblindness.

Concerning contemporary research, McIntire et al. (2014) investigated the effect of varying disparity on task performance for a virtual object precision placement task while viewing a stereoscopic display. The authors concluded that “clinically normal stereopsis does not ensure a performance benefit from stereoscopic 3D depth cues.” However, what they call clinically normal stereopsis was validated by the “Titmus stereovision test,” assessment of phoria, and vergence, which cannot confirm that their subjects were not stereoblind, affecting their conclusion. Indeed, some subjects, who passed the stereovision test, showed atypical responses. Bosten et al. (2015) set out to conduct a population study of binocular function in 1,060 participants, in which they, among many other visual functions, assessed near and far disparity sensitivity. However, to assess stereovision they used the TNO stereo test in which subjects are not restricted in making eye movements meaning that, again, a subset of their subjects may have been stereoblind, again most probably affecting their conclusion. Other recent work addressed human functional magnetic resonance imaging in human (Goncalves et al., 2015). They used a presentation duration of 1 second, and eye movements were not recorded; it is unclear how they objectively assessed the stereo abilities of their subjects. Similarly, another recent functional magnetic resonance imaging study (Nasr, Polimeni, & Tootell, 2016) examined activity in V2 and V3 to a mixture of far and near disparities. Without the results of the stereoblindness test, the results reported in these articles must be qualified. If the authors would have mapped performance in a proper stereoblindness test, interpretations would have been more convincing.

After 50 years of stereoblindness, there is still an open conundrum: How can the established finding that disparity is processed by a continuum of detectors be reconciled with the disability of many observers to process a whole class of far or near disparities? We propose, based upon integration of literature, that an asymmetry between far and near disparity detection at birth—possibly being present for a variety of reasons—can suppress the typical formation of binocular correlation during the critical period for the development of stereopsis early in life, thereby disabling a whole class of far or near disparities. This proposal is in line with the above reviewed findings: Hubel and Wiesel (1965) reported that kittens with artificially induced squint lacked binocular neurons, attributed to a lack of binocular correlation during the critical period for the development of stereopsis. In addition, Jones’ (1977) findings can be interpreted that stereoblindness could be caused by a disability to make correct fixational eye movements in depth early in life, in turn, causing a lack of exposure to binocular correlation during the critical period for the development of stereopsis. Moreover, a twin study has found that strabismus has a genetic aetiology (Wilmer & Backus, 2009). People with inherited strabismus have an asymmetric and unbalanced use of near and far disparities. This asymmetry may lead to a predominance of eye postures that enable the processing of far disparities more than the processing of near disparities, or vice versa. Thus, an asymmetry between far and near disparity detection at birth can naturally lead to suboptimal routine oculomotor behaviour that suppresses the typical formation of binocular correlation during the critical period in the development of stereopsis for a whole class of far or near disparities. Next to strabismus (that can still lead to the formation of binocular neurons if properly dealt with), there may be more severe genetic deficits: Retinal axon guidance involves a complex genetic pathway (Prieur & Rebsam, 2016), and retinal axon guidance defects have been frequently displayed in zebrafish (Hutson & Chien, 2002). Genetic defects influencing development of the human optic tract are, thus, not unlikely. Such genetic predisposition may lead to a failure to develop binocular neurons and may cause stereoblindness. In conclusion, an asymmetry between far and near disparity detection during the critical period in the development of stereopsis can cause suppression of a whole class of far or near disparities.
